# Muslim Women Inmates and Religious Practices: What Are Possible Solutions?

**DOI:** 10.3390/healthcare13151890

**Published:** 2025-08-02

**Authors:** Maria Garro

**Affiliations:** Department of Psychology, Educational Science and Human Movement, University of Palermo, 90128 Palermo, Italy; maria.garro@unipa.it

**Keywords:** Islam, Italy, Muslim women, prison

## Abstract

**Background/Objectives**: Despite legal frameworks acknowledging the need to protect the rights of female prisoners, penitentiary systems often neglect gender-specific needs, particularly for foreign women. Among them, Muslim women face distinct challenges linked to cultural and religious practices, which are frequently unmet in prison contexts. This review aims to explore the academic literature on the experiences of Muslim women in detention. **Methods**: A systematic review was conducted using three major bibliographic databases—Scopus, PubMed, and Web of Science—covering the period from 2010 to 2024. Inclusion criteria focused on peer-reviewed studies examining the condition of Muslim women in prison. Of the initial pool, only four articles met the criteria and were included in the final analysis. **Results**: The review reveals a marked scarcity of research on Muslim women in prison at both national and international levels. This gap may be due to their limited representation or cultural factors that hinder open discourse. The selected studies highlight key issues, including restricted access to services, limited ability to practice religion, and language and cultural barriers. These challenges contribute to increased psychological vulnerability, which is often underestimated in prison settings. **Conclusions**: There is an urgent need for targeted research and culturally competent training for prison staff to adequately support Muslim women in detention. Greater academic and institutional attention is essential to develop inclusive policies that consider the intersection of gender, religion, and migration, particularly in the post-release reintegration process.

## 1. Women in Prison: Between Regulatory Aspirations and Structural Limitations

The prison system’s structure has historically underestimated the specificities related to the female gender, leading to a systemic neglect of women in prison. Despite women representing a small percentage of the prison population, the penitentiary treatment reserved for them has not received adequate attention over time, especially in terms of application, despite the regulatory aspect appearing beneficial [1,2,3,4].

This article explores the tension between progressive regulatory efforts and the persistent structural limitations that continue to shape female imprisonment.

A decisive step towards starting a systemic reflection on the penitentiary treatment of women was represented by the adoption, in 2010, by the 193 member states of the United Nations, of the Rules for the Treatment of Women Prisoners and for Non-custodial Measures for Women Offenders, commonly known as the Bangkok Rules. This body of legislation consists of 70 provisions designed to fill the gaps in the Standard Minimum Rules for the Treatment of Prisoners (the so-called Mandela Rules), which date back to the 1950s and were built around a male prison model (www.unodc.org, accessed on 13 March 2025). The Bangkok Rules, therefore, are divided into two main sections: the first contains the principles of general application for all female prisoners, while the second is dedicated to particularly vulnerable categories, including mothers, foreign women, and young people. Although lacking binding force, the rules insist on considering the specific needs of women to avoid discriminatory treatment. It is no coincidence that some provisions are dedicated to foreign female prisoners, with particular reference to the transfer policies to their country of origin and the repatriation of their children (www.ohchr.org; www.unodc.org, accessed on 13 March 2025).

Formal adherence to the principles enshrined in the Bangkok Rules has not always been reflected in practice, as demonstrated by their violation in several countries [5] and the impossibility of or resistance to implementing them in others [6]. This results in a persistent gap between international standards and institutional practices, which risks nullifying the effectiveness of the rules themselves despite the presence of women prisoners at a global level.

The significance of the Bangkok Rules lies not only in their policy-oriented nature but also in their clear anchoring to international human rights frameworks. By addressing gender-specific needs and urging states to avoid discriminatory practices, these rules directly engage with key human rights principles, such as the right to equality before the law, the right to health, the prohibition of inhuman or degrading treatment, and the right to freedom of religion. These rights are enshrined in core international instruments, including the International Covenant on Civil and Political Rights (ICCPR), the Convention on the Elimination of All Forms of Discrimination against Women (CEDAW), and the European Convention on Human Rights (ECHR). As such, the lack of implementation of the Bangkok Rules in many countries can be read not only as a policy failure but also as a broader human rights concern.

In this sense, it is reported that, in February 2024, the percentage of women in prison was 1.2% in Albania, 2.8% in Azerbaijan, 3.7% in Bulgaria, 3.9% in Turkey, 4.5% in Denmark, 5.6% in Germany, 6% in Switzerland, 6.2% in Austria, 7% in Portugal, 7.1% in Spain, 8.1% in the Czech Republic, and 8.3% in Iceland. The data for the USA are dramatic, with 64.2 prisoners for every 100,000 women; this rate of female incarceration was 15 times higher than the Italian rate, as documented by Antigone (XXI Rapporto 2025, www.rapportoantigone.it, accessed on 13 March 2025). In fact, in Italy, as of 28 February 2025, women constituted 4.3% of the prison population, equal to 2729 prisoners out of a total of 62,165 people in prison (Available on: https://www.giustizia.it/, accessed on 13 March 2025). This is a “penitentiary minority” to which, despite the legal protections in its favor, adequate attention has not been paid in everyday life [7].

In fact, Law No. 354 of 1975—Rules on the penitentiary system and on the execution of measures depriving and limiting freedom—recognizes some aspects relevant to the protection of female prisoners, both about the need to ensure inspections and transfers by personnel of the same sex, as well as for their condition as mothers, present or future (Articles 11 and 39), and the need to host them “in separate institutions or in special sections of the institution” (Article 14). In practice, however, there are only four penitentiary institutions exclusively dedicated to women (Pozzuoli, Rome “Rebibbia,” Trani, Venice “Giudecca”). At the same time, in the rest of the country, female detention is entrusted to departments created within male prisons (approximately 45 sections).

Greater attention to the needs of females was subsequently presented in the new Regulation for the Execution of the Ordinance (Presidential Decree 30 June 2000, no. 230, which came into force on 6 September 2000). Article 8 introduces references to personal hygiene, Article 9 refers to clothing and equipment, and Article 34 underlines the need to consider female prisoners’ physical, professional, social, and psychological needs in every decision regarding their detention. These references contribute to outlining a complex female identity, which cannot be reduced to the biological dimension alone but extends to biographical and social elements. Finally, Article 81 provides that prison staff should be adequately trained to work with the female prison population, recalling the importance of specific preparation from an organizational and relational perspective. Despite these advances, the concrete implementation of these provisions has remained partial and uneven.

In 2008, the Department of Penitentiary Administration issued an internal regulation prepared for female sections that considers the particularities of gender-sensitive penal execution. This is to develop organizational measures and rehabilitation suitable for capturing and enhancing the specificity of the female prison population. Particular attention is paid to the affective dimension, specific health needs, the different relationships with one’s physical needs, the need to offer equal opportunities for social reintegration, and an increase in the occasions in which there is co-presence with male prisoners (school and training in general, cultural, recreational, and sports initiatives, participation in the representative commissions provided for by the penitentiary system) (Available on: https://www.giustizia.it/, accessed on 13 March 2025).

However, the female component of the prison population generally does not share spaces or activities with the male component, resulting in even more segregation within the prison environment. On the one hand, women in prison are separated from their families and places of residence. On the other hand, they are deprived of specific work, training, or even educational opportunities compared to their male counterparts (Available on: https://www.antigone.it/, accessed on 13 March 2025).

Female detention is a recognized issue in global prison policies, but it is often not given the attention it deserves. There is a persistent gap between the drafting of legislation and the actual implementation of gender-sensitive prison measures, indicating an institutional system that is still largely based on a male detention model. This system is not equipped to address the complex needs of female prisoners in a systemic and structural manner, as will be further highlighted below [7,8].

## 2. Muslim Women in Italian Prisons

Among those most affected by the structural limitations discussed so far are foreign and Muslim women, whose conditions in Italian prisons reflect multiple layers of marginalization. Although the presence of Muslim women of Italian origin, including converts, is acknowledged, the focus here is primarily on foreign Muslim women, whose specific vulnerabilities and greater visibility in the prison population make their situation particularly urgent and in need of attention.

According to data updated as of 28 February 2025, Italy had 767 foreign women prisoners, equal to 13.8% of the total number of foreign prisoners, which amounted to 5534 individuals (giustizia.it). The crimes most frequently attributed to foreign women were those against property (29.9%), followed by drugs (10.2%), and prostitution (5.4%), because they are often victims of trafficking. Finally, 4.9% of foreign women are in detention for resisting the orders of and insulting public officials (Available on: https://openmigration.org/, accessed on 17 March 2025).

These women, due to their poor or absent knowledge of the language and legal system of the host country, are at a heightened state of vulnerability. This condition makes them particularly exposed to marginalization and difficulty in accessing rights and services, both inside and outside the penitentiary institution. This lack of support from cultural mediators, who can assist them in dealing with bureaucratic and legal procedures, further complicates their path to defense and social reintegration [9].

In addition, there is a lack of specific data on the religious affiliation of foreign female prisoners, accentuating the difficulty of adequately satisfying their religious and spiritual needs, which is a fundamental aspect of not only their identity but also the treatment process.

Italy is a secular state, which implies it must remain neutral regarding religious beliefs without favoring or discriminating against any. The Italian Constitution (1946) guarantees everyone the right to freely profess their religious faith, individually or collectively, publicly or privately. All religious confessions are equally free before the law, and none can be subject to discrimination (art. 8). Equally important, no one can be forced to declare a religion (art. 19).

Finally, the General Data Protection Regulation (GDPR) classifies religious affiliation as “sensitive” personal data (art. 9), which means that it requires special protection due to its potential to cause harm or discrimination if misused.

For these reasons, in the penitentiary context, the available information regarding prisoners’ faith is mainly based on inferences related to their nationality, determining an incomplete or complex picture.

Following this criterion, therefore, as of 28 February 2025, the main nationalities represented among foreign women in detention in Italy are Romanian (n = 188), Nigerian (n = 91), Moroccan (n = 48), Bosnia-Herzegovinian (n = 37), Brazilian (n = 32), Bulgarian (n = 29) and Albanian (n = 24). These territories suggest that they may be of the Muslim faith. However, these data do not directly indicate religious affiliation (www.giustizia.it, accessed on 17 March 2025).

The religious aspect is important because it represents one of the main areas of conflict in Italian prisons for both women and men under the same conditions of detention. In the specific case of Islam, some practices—such as fasting during Ramadan, eating times, the need for clean water for ritual ablutions (wudu) before the five daily prayers, the observance of a halal diet, or the use of the veil (hijab) by women—may conflict with the material and organizational needs of the prison context.

These needs, linked to freedom of worship, often require specific adaptations that detention facilities cannot always guarantee. This lack of religious equality raises complex issues regarding respect for fundamental rights, highlighting the need for policy change. This represents a problem for the exercise of a principle, such as the freedom to profess one’s religion (art. 26 L.354/75), as recognized by the United Nations High Commissioner for Human Rights (www.ohchr.org, accessed on 17 March 2025).

In some Italian prisons, the administration tries to accommodate the needs of Muslim inmates. For instance, they offer food at special times to allow for fasting during Ramadan, or they allow the wearing of the hijab, which is a central element of their religious identity for many women. However, these initiatives are often local and not always sufficiently coordinated, leading to inconsistencies in the treatment of Muslim inmates across different prisons.

In the United States, for example, Muslim women make up 9% of the country’s state and federal prison populations. This number is growing, and with it, the instances of rights violations. Many incarcerated Muslim women have been denied the right to wear religious clothing when arrested or imprisoned. Some cases included a lack of access to prayer spaces, denial of food at specific times during Ramadan, and access to religious texts (Available on: https://19thnews.org/, accessed on 17 March 2025). The arrest process can also cause significant distress, especially for women who are asked to remove their headscarves for mug shots, which become public and are often published by newspapers or websites, causing shame, anxiety, stress, depression, and PTSD (Ibidem).

Therefore, this general reality constitutes a clear violation of the right to religious freedom [10], although religion increasingly plays a significant role in the rehabilitation of people in prison [11], and in forming communities of support and alliances within the group, protecting them from the harm of the prison environment [12].

The denial of such rights not only discriminates against foreign Muslim women in Italian prisons but also affects Italian female inmates who have converted to Islam, for whom the same obstacles to religious practice that must be faced by immigrant women can apply. The latter, however, are more exposed to a high level of marginalization dictated by the intersection of gender, religion, citizenship, and ethnicity, undermining their dignity and respect for their beliefs [13,14].

Added to this is the stigma against Islam in general since 11 September 2001 [15,16,17] (Available on: https://www.antigone.it/, accessed on 13 March 2025). A rigid attitude, noted in the West, against Muslims which, according to what was reported by researchers at the University of Leuven in the framework of the Horizon 2020 project “HumMingBird,” appears to be vigorously reinforced by the impact of the media—from television to digital newspapers—and by alarmism linked to migration, contributing to the birth of ethnic prejudice and hostility (Available on: https://hummingbird-h2020.eu, accessed on 13 March 2025).

## 3. Research Objectives

The ongoing debates and challenges concerning the visibility of Muslim women in prison, as well as their vulnerabilities highlighted thus far, have motivated the present study. This study aims to explore the existing academic literature on this topic, with reference to the Italian context. The goal is to enhance understanding of this phenomenon and better identify its specific implications within the psychological field. To achieve this, the review intends to address the following questions. What aspects of the presence of Muslim women in prison are highlighted in indexed publications? What are the consequences of incarceration faced by Muslim women? Additionally, what research has been conducted in Italy on this subject?

These interrogative statements indicate the intention to explore the insights provided by academic dissemination regarding Muslim women in detention and their needs.

## 4. Method

This review was conducted to provide a synthesis of scientific evidence on Muslim women in prisons in Italy and other countries. It follows the components of the Preferred Reporting Items statement of the PRISMA structured methodology (Preferred Reporting Items for Systematic Reviews and Meta-Analyses) [18,19,20,21].

For this purpose, the relevant literature in the psychological field was examined by searching the following databases: PubMed/Medline, ISI Web of Science (WOS), and Sage Journals for peer-reviewed scientific journals. During the preliminary phase, an exploratory consultation of the PsycINFO database was also carried out, but it did not return results relevant to the specific focus of the study. Similarly, the research conducted via Google Scholar identified several contributions that, once again, did not meet the established inclusion criteria, particularly regarding thematic relevance and methodological rigor.

The inclusion criteria for the selection of studies were as follows: (1) published between 2010 and 2024, (2) written in English, and (3) qualitative–quantitative studies on Muslim women in prison.

The year 2010 was chosen as an inclusion criterion due to its significance in marking the beginning of the Arab Spring, which brought attention to prison conditions in many countries. The timeframe of 2010–2024 was selected to ensure that the review is focused on recent studies, thereby incorporating the most current and relevant research on the topic.

The criterion regarding the language of the publications analyzed (English) was chosen because it is the main language in which a significant part of high-quality international scientific research is published and is widely accessible in many databases and repositories. This choice also restricts the search to a well-established set of studies, thus minimizing potential problems related to translation or interpretation, which could affect the reliability of the results (bias).

The exclusion criteria were (1) reviews, books, book chapters, letters to the editor, and gray literature; and (2) studies on both Muslim men and women confined as prisoners.

In particular, gray literature was excluded from the review, since recent, non-indexed publications that provided valuable contributions to the topic were considered in the development of this article. Therefore, each document was included in the text only after careful evaluation of its quality and relevance.

Therefore, to obtain relevant literature in academic databases in the psychological sector, the choice of keywords was oriented towards “women,” “Muslims,” “prison,” and “Italian,” interrelated with the Boolean operators “AND” and “OR”. These keywords were selected to ensure a comprehensive search that covers all aspects of the topic. After entering the search strings, the inclusion and exclusion criteria were set to obtain literature from the databases. For the potentially eligible publications, abstracts were read, and the full text was retrieved.

Academic ethical guidelines were not followed during the study to ensure consent acquisition, anonymity, confidentiality, and data management, since no participants were involved, and the data used could be traced in the databases.

## 5. Results

**Figure 1:**
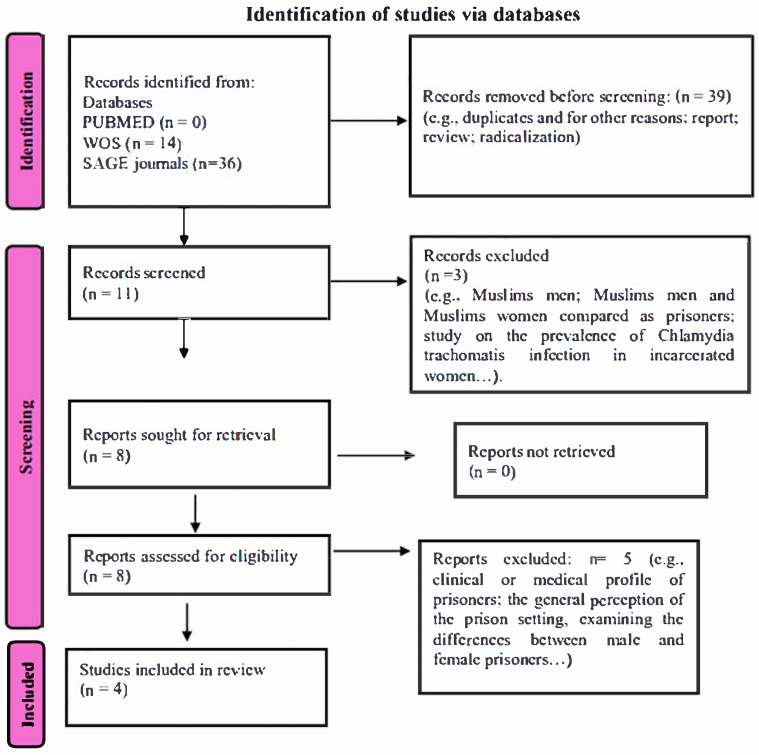
Flowchart of the process of including articles in this review.

Through databases and manual searches, 50 records were identified. After 39 duplicates and reports were removed, 11 titles and abstracts were screened, and eight full-text articles were assessed for eligibility. Four articles met the inclusion criteria and were considered most suitable for the review (see Figure 1). The following information was extracted from each article included in the review: authors, title, publication year, abstract, and general results (Table 1).

The following sections discuss and organize the results derived from the analysis of articles related to the areas of interest according to the review’s three research questions.

To better illustrate the methodological approaches and thematic aspects shared by the included studies, it was deemed appropriate to develop a comparative matrix. Table 2 highlights the data collection tools, key themes identified in each study, and overlapping thematic connections, providing a clearer summary beyond the descriptive analysis.

This review was undertaken to answer the following questions. What, if any, indexed publications focus on the presence of Muslim women in prison? What are the effects of detention on the well-being of this specific portion of the prison population? What studies have been conducted in Italy?

### Selected Articles

The studies included in this review, published in indexed journals during the period considered here (2010–2024), aimed to significantly contribute to the visibility of Muslim women’s experiences in prison and to their relationship with their own identity, culture, gender, and religion.

a. Studies focused on Muslim women in prison.

The contribution of Castillo-Algarra and Ruiz-García (2024) [22] has the merit of having supposedly been the first to emphasize the relationship between the Muslim religion and prison and between religion and women in jail, within a gender perspective. The authors do not hesitate to highlight the different perspectives of the Muslim religion and the multiplicity of interpretations of Islam existing in Morocco, depending on the country’s area, as well as their influence on societies and the role of women in them. For this reason, to know how and to what extent the Moroccan prisoners practice their religion and the role that it plays for them in prison, it is necessary to identify their places of origin, understand how they have interpreted Islam in their communities, and how Islam affects their lives as women and prisoners (ibid.). In this sense, the results of the contribution by Gómez et al. (2022) [24] regarding jihadists should also be read to understand the motivation behind the low levels of self-esteem and resilience. The specific needs and characteristics of jihadist prisoners, the authors say, should be taken into account in both the development of treatment programs and the use of risk assessment tools—a subject also extremely relevant but almost unexplored.

Obviously, these aspects demand the multicultural competencies of the penitentiary operators and the participatory involvement of the accredited ministers. Therefore, the training of practitioners, the creation or strengthening of the secondary support network, and the active involvement of local Islamic communities are key resources to promoting the well-being of Muslim women in detention.

b. Implications of detention on the well-being levels of Muslim women.

Subjective well-being, understood as cultivating and expressing individual virtues in harmony with the surrounding world [26], is protected through religion. Jodi et al. (2015) [25] conclude their contribution by stating that the cause of the social problems faced by all Muslim prisoners is to be attributed to a lack of religious knowledge, as well as factors of socio-economic instability and modern lifestyle. The authors, therefore, focus on the need to follow the halaqa program, which is traditional Islamic pedagogy [25,27]. However, the prison context does not always consider the spiritual needs of the audience. Furthermore, Einat and Chen (2012) [23] point out that one of the ways to harmonize with the prison environment can be achieved is the creation of prison family networks, which may represent an “adequate substitute” for biological and/or normative families. These family structures help inmates to cope with the “sorrows of captivity effectively” and constitute a significant behavioral control mechanism, alleviating stress and depression [23]. In addition, pseudo-family networks in women’s prisons reflect gender cultural expectations and are a way for inmates to maintain their identity and self-esteem. These relationships meet inmates’ social, economic, psychological, recreational, and physiological needs, hence the dimensions of well-being [28]. At the same time, they enable them to respond to social beliefs about gender roles.

c. Studies on Muslim women in Italian prisons.

The academic literature on the relationship between Muslim women and prison experience in Italy is seriously lacking. Indeed, the few studies dedicated to Islam within the Italian penitentiary system, while offering valuable perspectives on the intersection of religion, culture, and detention, shed light on the unique experiences of Muslim prisoners [29,30,31,32], but they do not devote space to the specific theme of Muslim women. The fact that so little is known about Muslim women in prison may be due to the relatively low number of inmates, who risk being perceived as unworthy of attention by the community and agencies. Furthermore, as mentioned above, under Italian law, it is not permitted to collect information on individuals’ religious beliefs, even in contexts such as prison.

This makes it particularly difficult to systematically identify and analyze the experiences of Muslim women in prison.

## 6. Discussion

This review examined articles focusing on the condition of Muslim women in detention, in countries other than their country of origin, to fill the multiple gaps in research, including publications dedicated to (1) the presence of Muslim women in prison, (2) the effects of detention on the individual well-being of Muslim women in detention, and finally, (3) studies on Muslim women in Italian prisons.

In terms of the overall results of the review, considering the studies included, the analysis highlights the substantial gap in the scientific literature concerning Muslim women prisoners, with particular reference to the Italian context.

The literature, composed mainly of qualitative studies, significantly contributes to understanding the experiences lived by the subjects involved. However, the results are still limited in number and scope. The qualitative approach is suitable for analyzing complex and multidimensional phenomena, especially when modelled by intersectional factors such as religion, ethnicity, and gender. The choice of qualitative drawings also responds to the difficulty of finding large and representative samples, thus making the results difficult to generalize but still relevant for exploring subjective dynamics [33] (www.apa.org, accessed on 13 March 2025).

This approach is practical when the variables to be explored are not easily quantifiable or when individual experiences play a key role [34]. The experience of a Muslim woman in prison is influenced by multiple factors, such as religion, ethnicity, gender, and the dynamics of the prison system itself.

In addition, studies generally highlight the central role of spirituality in daily life in prison. In this sense, the difficulties encountered in practicing it translate into a negative impact on psychological well-being, as defined by the eudaimonic model of Ryff (1989) [28].

This is also confirmed by Purdie et al. (2021) [35], who, based on data collected in ten prisons, including two male and female prisons, one in England and the other in Switzerland, show that prison tends to intensify the religiosity of Muslim men and reduce that of Muslim women. To explain this, the authors argue that, at an individual level, guilt about the absence of family, high-ranking gendered religious forms, and feelings of trauma and victimization hurt the religiosity of Muslim prisoners.

According to the authors, women’s religious status is influenced by their minority status in prison; therefore, they have less influence as a community and are more likely to suffer from blatant discrimination by staff and other inmates [35], which, as a result, affects their well-being.

Finally, the Italian case is particularly critical. The absence of specific studies on Muslim women in the penitentiary system indicates a knowledge gap that may result in a lack of response from institutions and inclusion policies. It is therefore necessary to promote research, with methodological rigor, which will explore in depth the needs of this vulnerable group to ensure the effective protection of their rights, encourage sustainable reintegration paths, and respect cultural and religious differences.

These, in conclusion, are the objectives of the national project PRIN2022 “Islam and Muslims in Italy: Actors, Social Space and Relations between Religious Communities and the State” (October 2022–February 2026), funded by the Italian Ministry of Universities and Research (PRIN 2022 Project 20228R992T: “Islam and Muslims in Italy: Actors, Social Space and Relations between Religious Communities and the State” PNRR—Mission 4: Education and research, Component C2: “From research to business”, Investment 1.1 “Fund for the National Research Programme and Projects of Significant National Interest (PRIN)” financed by the European Union—NextGenerationEU). The study presented here is, in fact, a deepening of the project topic, which arises from the observation of a gap in academic literature on the subject. The four partner universities (Palermo, Padova, Verona, and Bari) are engaged in research activities, scientific dissemination, and dialogue with civil society with the aim of raising awareness on the subject and promoting informed and participatory change (https://islamitaly-prin-mur.eu/, accessed on 17 March 2025). In this context, public events were organized, such as the World Café (Turin, 2025) and discussions and special dialogue with institutions and citizens (Palermo, May 2025), as well as participation in national and international scientific conferences.

## 7. Limitations and Future Research

This review has limitations. Firstly, the restriction to English-language publications inevitably narrowed the scope of the analysis, especially given the prominence of the Italian context, where relevant research is often published in Italian or other languages. Consequently, some significant studies may have been excluded, limiting the comprehensiveness of the review.

Secondly, due to the nature of the topic, some relevant publications may have been inadvertently excluded on this occasion, due to time constraints or the nature of the articles. Thirdly, the inclusion criteria for relevance were not limited to specific geographic regions and contextual sizes, and access to data is difficult due to cultural norms that affect data communication. Another problem is the heterogeneity of the studies analyzed and the low number of samples, which limits the possibility of generalizing the results. Finally, identifying specific sub-groups, such as converted Italian women, remains problematic.

Therefore, the review’s development should plan to analyze a wider range of inclusion criteria, avoiding overly focused approaches.

Despite these limitations, the review presents a synthesis of studies showing evidence capable of prompting new research. The importance of supporting initiatives such as research, training programs for prison operators, and integrated interventions to protect the well-being of women prisoners in general, and Muslim prisoners in particular, must be reiterated. These measures are essential to promoting a deeper understanding of the subject and awareness of the often-mentioned intersection between gender, ethnicity, and religion.

## 8. Conclusions

National and international legislation, such as the 2010 UN Bangkok Rules, recognizes the need for differentiated and respectful treatment of gender and cultural diversity. But the reality of Muslim women in prison still highlights a significant gap in penitentiary policies, at the global level, as well as the interest in the experience of Islamophobia of Muslim women in prison [11] (www.meforum.org; www.russellwebster.com/invisible-muslim-women/, accessed on 13 March 2025).

The collection of data from a sample of Muslim women in prison, hampered by logistical issues such as access to prisons or the reluctance of the inmates themselves to participate in research development, should encourage understanding of the specific needs of this particular category of detainee. Thus, the promotion of studies, for example, on the differences through which Islam is experienced in prison: the devotion of Muslim prisoners tends to be high, but women need a gender-sensitive chaplaincy [36]. It seems useful, therefore, to reinvent religious programming to avoid a “one size fits all” model [37], also because following migration, religion helps women manage stress (coping) [38].

Therefore, the Muslim Women in Prison Project (MWiP) (2013), based at the Khidmat Centres (Bradford), specializes in providing personalized support to Muslim women in prison. Sofia Buncy, National Coordinator, has been instrumental in raising awareness among professionals in the criminal justice system (CJS) and local communities about the specific difficulties Muslim women face in prison [39] (www.criminaljusticealliance.org, accessed on 13 March 2025). From this experience, the documentary *Inside Out* was produced, which is also aimed at developing culturally appropriate and equitable support models (Available on https://www.criminaljusticealliance.org/, accessed on 14 April 2025).

In Pakistan, similarly, the state’s need to meet the basic needs of women in prison is recognized, even though they have insufficient facilities and are not granted fundamental human rights [40]. Some more of the common goals of “Project Fatima” (www.taybafoundation.org, accessed on 13 March 2025), or Muslim Hands, the UK-based international Muslim charity, which commissioned the Huddersfield Pakistani Community Alliance (HPCA) to undertake an assessment of the problem and identify specific areas of need for Muslim women (https://muslimhands.org.uk, accessed on 13 March 2025), include addressing the tendency of the Muslim community to accept male prisoners and to marginalize and label women as bearers of shame and dishonor for the family and community (ibid.).

This reality urges identification of suitable strategies for post-prison social reintegration, also because, as Joly (2016) [41] points out, the results of research conducted in Great Britain found that women in Muslim communities can develop differentiated strategies concerning the objectives pursued and the enabling factors they encounter in the host country. In this sense, it is possible to affirm the expansion of their presence and participation in the public sphere of the majority society. This is an indication of their possible autonomy and ability to act on behalf of the communities to which they belong, which, if exploited, can help reduce ostracism and recidivism of former prisoners.

Finally, within the framework of training for prison operators, it is necessary to understand and consider the context of experiences, concerns of women in detention, as well as the local and national focus on the treatment of individuals belonging to minority groups [42].

Already included in the Bangkok Rules (rules 29–35) is an important chapter on prison staff, whose specific professional training becomes the main instrument, at all levels, to put in place the necessary measures to meet gender-specific needs and eliminate discriminatory practices against women (https://www.unodc.org/, accessed on 13 March 2025).

It is also widely recognized that training is a determining factor in improving the well-being levels of healthcare professionals and users, since it acts as an empowerment tool [43,44]. Indeed, through appropriate training, it is possible to promote the quality of life of individuals, enhance their capacity for social interaction, and support the development of relational and emotional skills essential for an overall and lasting well-being [45,46].

In Italy, training programs have been launched to raise awareness among practitioners on issues such as the management of religious diversity, e.g., the project PriMED—Prevention and Interaction in the Trans-Mediterranean Space, funded by the Ministry of Universities and Research (2022); this course was designed for Muslim religious workers active in Italian prisons (https://primed-miur.it/, accessed on 13 March 2025). The PriMED project has laid the foundations of the above-mentioned project, Islam and Muslims in Italy, within which this review is inserted. The course on religious pluralism addressed prison officers in Lombardy, promoted by the Regional Board of the Penitentiary Administration in collaboration with Caritas Ambrosiana, COREIS Italiana, the Jewish Community of Milan, Catholic University, and the City of Milan.

To these are added initiatives dedicated to the prevention of radicalization in prison. Notably, the European project Train Training, developed in collaboration with the Catholic University of the Sacred Heart (www.transcrime.it, accessed on 13 March 2025), is worth mentioning. Finally, the Italian Penitentiary Administration has initiated the issuance of calls for the selection of cultural mediators to be employed in penitentiary institutions, aiming to strengthen the social inclusion of inmates of foreign nationality. Unfortunately, however, these initiatives do not involve all Italian penitentiary facilities.

Therefore, the welfare of women prisoners, both foreign and indigenous, and in general of the inmate population beyond gender, ethnicity, etc., can be effectively protected through adequate and widespread training of prison workers, which is not limited to limited interventions or pilot projects. An integrated approach should aim to promote the autonomy of prisoners, improve the quality of interpersonal relationships, and facilitate the interaction of individuals with social norms and institutions, thus helping to limit the negative impact of punishment.

## Figures and Tables

**Table 1:** Articles focusing on Muslim women in prison included in the review (alphabetical order).

Author(s)	Title	Topic	Participants	Results
Castillo-Algarra, J.; Ruiz-García, M. (2024) [22]	The Islamic religion in prison and Moroccan women prisoners in Spain	The main aim of this paper is to investigate how Moroccan women prisoners interpret and practice their religion in prison and the consequences of this in their lives, on an individual and group level—in short, the role religion plays for them in prison.	23 Moroccan women and 20 members of prison staff (Spain)	The research shows that the Muslim religion, as well as being one of the principal factors in helping Moroccan prisoners adapt to prison life, is also the principal element that differentiates them, in that the degree of influence which their faith exercises over them determines their identity both as women and as prisoners. The topic has not been investigated before, which represents the strongest point of this research, and at the same time, one of its limitations is that no study has been published on this topic before.
Einat, T.; Chen, G. (2012) [23]	What’s love got to do with it? Sex in a female maximum-security prison	Israeli female inmates’ attitudes toward same-sex sexual relationships in prison and incentives to participation.	46 adult inmates of a single Israeli female prison (Neve Tirza)	This study highlights a key finding regarding the relationship between ethnicity and attitudes toward same-sex sexual relationships in prison. Although most Jewish and Muslim female inmates expressed negative views on these relationships, the majority—90% of Jewish and 81% of Muslim participants—admitted to having engaged in them at least once. This suggests that ethnicity has little influence on actual behavior and plays only a minor role in same-sex sexual conduct within the current Israeli female prison subculture.
Gómez, Á.; Chiclana, S.; Chinchilla, J.; Blanco, L.; Alba, B.; Bautista, H.; Pozuelo-Rubio, F. (2022) [24]	The mirage of the jihad. Disenchantment as the pathway to disengagement of female jihadists. A case study about radicalization in Spanish prisons	The interest in disentangling the pathways to violent radicalization is one of the main areas of attention nowadays. However, investigations including data from terrorists are marginal, particularly in the case of women.	25 Muslim female inmates incarcerated in six Spanish prisons because of jihadist terrorism, or for crimes unrelated to terrorism	According to the findings, women who identify as jihadi engage in the process of radicalization because of a crisis in their personal and social identity. Still, they also seem to distance themselves from jihad due to disillusionment with unfulfilled expectations.
Jodi, M.K.H.; Mohamad, M.A.; Mohamad, M.T. (2015) [25]	The effectiveness of religious programme: Analysis of spirituality programme in prison among Muslim female inmates	The paper attempts to examine the prison institution in an effort to identify an Islamic-based program through a program that caters to halaqah modules in the prison. The halaqah program is an approach to treating and rehabilitating inmates so that they have the opportunity and space to transform themselves from immoral to morally behaved people.	Five inmates within the age range of 21–50 were chosen as research respondents from the Women’s Prison of Kajang, Selangor (Malaysia)	The study shows that the halaqah program is the core treatment for Muslim inmates. The success of this program is based on five modules, namely faith (God-consciousness); module of fiqh that emphasizes prayer as a source of peace; module of the Holy Quran as a prerequisite to worship; module of hadith nourishing a sense of responsibility; and the module of the Sirah of Muhammad and the Rashidun Caliphs Abu Bakr and Umar al-Khatab as guidance to face any obstacles in life.

**Table 2:** Comparative matrix of methodologies and common themes.

Author(s)	Data Collection Tool	Main Themes Identified	Shared Themes
Castillo-Algarra, J.; Ruiz-García, M. (2024) [22]	interviews	Role of Islam for Moroccan women in prison	Similar to Jodi et al.: Gender, religion as coping and identity; adaptation and transformation in prison
Einat, T.; Chen, G. (2012) [23]	semi-structured interview	Same-sex relationships in prison	Similar to Castillo-Algarra and Ruiz-García: Gender, cultural norms, and contrast between religious values and prison behavior
Gómez, Á.; Chiclana, S.; Chinchilla, J.; Blanco, L.; Alba, B.; Bautista, H.; Pozuelo-Rubio, F. (2022) [24]	interviews	Radicalization and disengagement	Similar to Jodi et al.: Identity crisis and personal transformation; role of religion in both radicalization and disengagement
Jodi, M.K.H.; Mohamad, M.A.; Mohamad, M.T. (2015) [25]	interviews and observations	Halaqah religious rehabilitation program	Similar to Castillo-Algarra and Gómez et al.: Islam as central to adaptation, rehabilitation, or disillusionment in prison

## Data Availability

The datasets generated during the current study are available from the corresponding author upon reasonable request.
